# Thermostable Esterase from Thermophilic *Laceyella sacchari*: Gene Identification, Heterologous Expression, and Biocatalytic Characterization

**DOI:** 10.3390/genes16111330

**Published:** 2025-11-03

**Authors:** Yu-Pei Chen, Xingru Zeng, Hsuan-Jung Peng, Ching-Yu Tu, Min Tseng, Li-Ling Liaw, Hongtan Wu, Fangfang Chen, Yang-Cheng Kuo

**Affiliations:** 1 The School of Public Health and Medical Technology, Xiamen Medical College, Xiamen 361023, China; wht@xmmc.edu.cn (H.W.); 200700010183@xmmc.edu.cn (F.C.); 2 Engineering Research Center of Natural Cosmeceuticals College of Fujian Province, Xiamen Medical College, Xiamen 361023, China; 13615980701@163.com; 3 Bioresource Collection and Research Center, Food Industry Research and Development Institute, HsinChu 300, Taiwan; phj@firdi.org.tw (H.-J.P.); tcy@firdi.org.tw (C.-Y.T.); t261215@gmail.com (M.T.); lll@firdi.org.tw (L.-L.L.)

**Keywords:** esterase, *Laceyella sacchari*, genomic shotgun library, p-NP butyrate, thermostability

## Abstract

Background/Objectives: Esterases are widely used in various industrial fields. This study aimed to isolate and characterize esterase genes from *Laceyella sacchari* HS49-1, a thermophilic bacterium from a hot spring, which can survive at 45–60 °C and pH 5-10 with robust esterase activity. Methods: A genomic shotgun library was constructed to identify three esterase genes: two in family XII (Est2 and Est7) and one in family VIII (Est1). Sequence analysis revealed significant divergence from other genera. Only Est1 was successfully expressed in *Escherichia coli*. Its activity, optimal conditions, thermostability, and structure were investigated using p-NP butyrate, temperature/pH assays, heating pre-treatment, and fluorescence quenching. Results: Est1 demonstrated high activity (57.43 ± 0.04 U/mg) towards short-chain p-NP butyrate (C4). Molecular-docking analyses revealed that Est1’s catalytic motif (GXSXG) interacts with various p-NP esters, with binding energy and interaction types varying by acyl chain length. The optimal temperature was 60 °C, and the optimal pH was 8. Est1 exhibited excellent thermostability, retaining 90% of its activity after pre-treatment at 50 °C for 8 h and 69.8% after pre-treatment at 80 °C for the same duration. Fluorescence quenching showed that after 1 h at 80 °C, the fluorescence was reduced by only 16.6%, indicating remarkable heat resistance. Additionally, Est1 did not require metal ions as cofactors and maintained 74.8% of its activity in the presence of 0.1% SDS. Conclusions: The unique properties of Est1 from *L. sacchari* HS49-1 highlight its potential for industrial applications. Further exploration of this thermophilic bacterium could uncover more valuable genes.

## 1. Introduction

Lipolytic enzymes play a pivotal role across a spectrum of industrial sectors, including food processing, bioremediation, pharmaceuticals, detergent manufacturing, biodiesel production, and waste management [1]. The market for microbial esterases, especially lipases, was expected to be valued at $684.55 million in2025 and is projected to grow to $963.71 million by 2030 [2]. These enzymes, which are capable of hydrolyzing carboxyl esters of varying acylglycerol chain lengths, are categorized into two primary groups based on their enzymatic activities: triacylglycerol acylhydrolases, commonly referred to as lipases (EC 3.1.1.3), and carboxylic-ester hydrolases, known as esterases (EC 3.1.1.1) [3]. In the classification of lipolytic enzymes, there are 35 distinct families have been recognized. Among these, family I, which encompasses 11 subfamilies, is distinguished as the true lipases, while families II through XXXV are classified as esterases [4]. Microbial esterase, which primarily belong to the α/β-hydrolase family, are characterized by a conserved catalytic triad consisting of serine, histidine, and aspartic acid [5,6]. This structural feature is responsible for their broad substrate specificity and high catalytic efficiency.

Various esterases have been identified from diverse microbial sources. For instance, EstGoM, an autotransporter esterase derived from a marine *Pseudomonas* sp., exhibited the high hydrolytic activity toward *p*-nitrophenyl octanoate, with optimal activity at pH 9 and 60 °C [7]. Moreover, EstGtA3, an esterase from the thermophilic bacterium *Geobacillus thermodenitrificans* NG80-2, preferred short-chain substrates and exhibited peak activity at 60 °C [8]. Esterases derived from hyperthermophiles, such as *Thermotoga*, *Thermoanaerobacter*, *Aeropyrum*, *Archaeoglobus*, *Pyrococcus*, and *Sulfolobus*, demonstrated exceptional thermostability, with optimal temperature ranges for activity spanning from 60 to 100 °C [9]. These enzymes prefer short- and medium-chain *p*-nitrophenyl (*p*-NP) esters as substrates. These findings underscore the versatility of microbial esterases in degrading diverse substrates and highlight the potential of these thermostable esterases for industrial applications.

The genus *Laceyella* currently comprises five validated species (*L. sacchari*, *L. putida*, *L. sediminis*, *L. tengchongensis*, and *L. thermophila*) according to the NCBI database [10,11,12,13], which were originally classified under Thermoactinomyces before taxonomic reclassification [14]. These bacteria, which are Gram-positive, thermophilic, spore-forming, and aerobic, belong to the family Thermoactinomycetaceae. Their thermophilic characteristics have prompted extensive studies on various heat-resistant proteins from this genus, demonstrating significant biotechnological potential. Thermitase from *L. sacchari* DSM 43353 exhibits thermostable endo-protease activity, maintaining stability at 50 °C with functional activity across pH 6–11 [15]. A poly(L-lactide)-degrading serine protease from *L. sacchari* LP175 shows optimal activity at 60 °C and pH 9.0 [16], while β-1,3-glucanase from *L. putida* FM3001 displays optimal curdlan hydrolysis at pH 4.2 and 80 °C [17]. Additionally, L-leucine dehydrogenase from *L. sacchari* catalyzes reversible oxidative deamination/reductive amination of branched-chain L-amino acids with optimal activity at 60 °C [18], and α-amylase from *Laceyella* sp. DS3 functions optimally at 55 °C and pH 6–7 [19]. These studies collectively demonstrate that proteins derived from this genus maintain remarkable activity and stability under elevated temperatures, highlighting their substantial potential for industrial applications.

In this study, we successfully isolated a thermophilic bacterial strain from the hot spring of Taiwan, which was taxonomically classified as *L. sacchari*. This strain was deposited in the Bioresource Collection and Research Center (BCRC), Taiwan, with the accession number BCRC AC10098. Genomic analysis of the strain revealed the presence of esterase genes, and we constructed a genomic library to screen for genes encoding highly active esterases. The subsequent heterologous expression of the identified esterase gene in *Escherichia coli* allowed for the purification and biochemical characterization of the recombinant enzyme, which we designated as Est1. Notably, Est1 exhibited remarkable thermostability, retaining substantial catalytic activity even at extremely high temperatures.

## 2. Materials and Methods

### 2.1. Isolation, Cultivation, and Identification of Strain HS49-1

Samples of sediment and water were collected from the Tenguxi hot spring in Yilan County, Taiwan (24.5550684° N, 121.5159281° E). Approximately 100 mL of water and 10 g of sediment were collected in sterile polypropylene tubes, placed in insulated containers to maintain ambient temperature, and processed within 12 h after sampling. To isolate thermophilic actinomycetes, 1 g of sediment (or 1 mL of water sample) was first enriched in 20 mL of yeast extract–malt extract (YM) broth (yeast extract 4.0 g/L, malt extract 10.0 g/L, dextrose 4.0 g/L) and incubated at 45 °C for 24 h under aerobic conditions with gentle shaking. After enrichment, the culture was serially diluted (10^−1^–10^−6^) with sterile saline solution (0.85% NaCl), and aliquots were spread onto YM agar plates (YM broth supplemented with 15.0 g/L agar). Plates were incubated at 45 °C for 3–5 days under aerobic conditions. Multiple isolates exhibiting distinct morphology were obtained and screened for esterase activity using 4-nitrophenyl butyrate as the substrate. Among these isolates, strain HS49-1 exhibited the highest esterase activity and was therefore selected for further physiological and biochemical characterization. Optimal cultivation conditions of strain HS49-1 were determined by growing the isolate in YM medium under various temperatures and pH conditions. Enzymatic profiling was performed with the API ZYM kit (bioMérieux, Marcy-l’Étoile, France) at both 37 °C and 55 °C, and reactions were identified visually according to color development. Genomic DNA was extracted from HS49-1, and 16S rDNA was amplified with universal primers 27F (5′-AGAGTTTGATCMTGGCTCAG-3′) and 1492R (5′-GGTTACCTTGTTACGACTT-3′), followed by Sanger DNA sequencing. A phylogenetic tree was constructed in MEGA X using the neighbor-joining algorithm with 1000 bootstrap replicates to assess node reliability.

### 2.2. Genomic Sequencing of L. sacchari HS49-1

The genomic sequencing of *L. sacchari* HS49-1 was conducted by Genomics Bioscience & Technology Co., Ltd. (New Taipei City, Taiwan). DNA sequencing was performed using the Illumina MiSeq platform (Illumina, San Diego, CA, USA). Raw sequencing reads were processed by Trimmomatic to remove adapter sequences and low-quality bases [20]. Subsequently, de novo assembly was carried out using the Shovill pipeline, version 0.9 (https://github.com/tseemann/shovill) (accessed on 31 October 2022). Gene prediction and annotation of the assembled genome were analyzed with Prokka, resulting in multiple predicted protein-coding sequences [21]. Functional annotation of the predicted proteins was performed using COG (Clusters of Orthologous Groups) [22], GO (Gene Ontology) [23], and KEGG (Kyoto Encyclopedia of Genes and Genomes) [24,25] databases to elucidate protein functions and metabolic pathways.

### 2.3. Construction of a Genomic Shotgun Library from L. sacchari HS49-1

Genomic DNA was cloned using the pEZSeq™ Blunt Cloning Kit (pEZSeq Amp, Lucigen, now Biosearch Technologies, Hoddesdon, UK). DNA was sheared to 1–4 kb fragments with a Hydroshear instrument (BST Scientific, Rockville, MD, USA), size-selected on a 1% agarose gel, and purified by gel extraction. The recovered fragments were quantified with a NanoDrop spectrophotometer (NanoDrop ND-1000, Thermo Fisher Scientific, Inc., Waltham, MA, USA), end-repaired, and ligated into the pEZSeq Amp vector. Ligation products were electroporated into *E. coli* EPI300™ (Epicentre, now Illumina, Madison, WI, USA) competent cells. Transformed cells were plated on LB agar containing 4 mM IPTG, 40 µg mL^−1^ X-Gal, 10 µL mL^−1^ triolein, and 100 µg mL^−1^ ampicillin and then incubated overnight at 37 °C. Positive clones exhibiting esterase activity (clear hydrolysis zones) were selected for DNA sequencing. These clones were designated TS1, TS2, and TS7. The esterase-encoding genes derived from the TS1, TS2, and TS7 clones were designated *Est1*, *Est2*, and *Est7*, respectively. A phylogenetic tree of the esterases was constructed with the neighbor-joining algorithm implemented in MEGA X, using 1000 bootstrap replicates to assess node reliability.

### 2.4. Construction of Esterase Expression Plasmids

Gene synthesis and expression vector construction were commissioned to Azenta Life Sciences (Suzhou, China). The genes *Est1*, *Est2*, and *Est7* were subcloned into the pET15b expression vector without any codon optimization. The pET15b plasmid was first linearized with *BamHI*. The synthetic genes *Est1*, *Est2*, and *Est7* were then individually joined to the linearized plasmid using the Gibson assembly method [26]. The 5′ and 3′ ends of the synthetic genes were designed with homologous sequences to the pET15b plasmid, which are CCTGGTGCCGCGCGGCAGCCATATGCTCGA and GCTGCTAACAAAGCCCGAAAGGAAGC, respectively. Each insert gene was in-frame with the vector’s coding sequence. All expression plasmids were verified by full-length DNA sequencing to confirm both sequence accuracy and correct reading frame.

### 2.5. Overexpression and Identification of Esterases in E. coli

The expression plasmid was introduced into *E. coli* C43(DE3) competent cells by heat-shock transformation. Transformed cells were plated onto LB agar containing the ampicillin and incubated at 37 °C for 12–16 h until single colonies appeared. A single colony was then transferred to 5 mL LB medium supplemented with ampicillin and cultivated at 37 °C with shaking for 8–12 h to obtain an overnight culture. The starter culture was inoculated into 250 mL fresh LB medium in a 500 mL conical flask and grown at 37 °C with shaking at 175 rpm (THZ-98C, Shanghai Bluepard instruments Co., Ltd., Shanghai, China) for 3–4 h until the OD_600_ reached 0.4–0.6. Protein expression was induced by adding IPTG to a final concentration of 0.5 mM, and cultivation continued with shaking at 175 rpm (THZ-98C) for 12–16 h at 25 °C. After induction, cells were harvested by centrifugation, the supernatant was discarded, and the pellet was resuspended in Native Buffer (25 mM Tris-HCl, 150 mM NaCl, pH 7.0). The suspension was kept on ice and lysed by sonication. The lysate was clarified by centrifugation, and 10% (*v*/*v*) glycerol was added to the supernatant, which was stored at −20 °C until further use. This procedure was performed in triplicate.

After SDS-PAGE, proteins were transferred to a PVDF membrane at 4 °C by wet electroblotting. The membrane was immediately immersed in 20 mL of blocking solution (5% non-fat milk in TBST) and incubated for 1 h at room temperature with gentle shaking. Following blocking, the membrane was incubated overnight at 4 °C with gentle agitation in 4 mL of the same blocking buffer containing anti-6 × His Tag mouse monoclonal antibody (BBI Life Sciences, Shanghai, China) diluted 1:4000 (1 µL antibody stock in 4 mL buffer). The next day, the membrane was washed three times with PBST (5 min per wash). It was then incubated with HRP-conjugated goat anti-mouse IgG secondary antibody (1:4000 in blocking buffer) for 1 h at room temperature with shaking. After another three PBST washes (5 min each), equal volumes of ECL-A and ECL-B substrates from the SuperSignal™ West Pico Kit (Thermo Fisher Scientific, Waltham, MA, USA) were mixed and applied to the membrane. Following a 1-min incubation in the dark, chemiluminescence was captured using a Gel Doc XR+ imaging system (Bio-Rad, Hercules, CA, USA).

### 2.6. Recombinant Esterase Proteins Purification

Protein purification was performed using a Ni-NTA column according to the manufacturer’s protocol (Ni-NTA Purification System; Thermo Fisher Scientific, Inc., Waltham, MA, USA). The supernatant obtained after sonication was mixed with Ni-NTA resin and incubated for 3 h at 4 °C with gentle agitation to allow His-tagged protein binding. The resin was allowed to settle, and the flow-through was carefully discarded. The column was then washed five times with 15 mL Native Wash Buffer (50 mM NaH_2_PO_4_, 0.5 M NaCl, pH 8.0, supplemented with 20 mM imidazole, pH 6.0); after each wash the resin was allowed to sediment and the buffer was drained. Bound protein was eluted with 10 mL Native Elution Buffer (50 mM NaH_2_PO_4_, 0.5 M NaCl, pH 8.0, containing 250 mM imidazole, pH 6.0). The eluate was immediately loaded into a Vivaspin^®^ 6 ultrafiltration concentrator (Sartorius AG, Göttingen, Germany), concentrated by centrifugation, and collected. Glycerol was added to a final concentration of 10% (*v*/*v*), and the concentrated protein was stored at −20 °C.

### 2.7. Enzymatic Activity Assay of the Esterase

Esterase activity was assayed spectrophotometrically using *p*-nitrophenyl (*p*-NP) esters as substrates. Substrates with different acyl-chain lengths were selected, including short-chain *p*-NP butyrate (C4) and *p*-NP valerate (C5); medium-chain *p*-NP caprylate (C8) and *p*-NP caprate (C10); and long-chain p-NP laurate (C12), *p*-NP myristate (C14), and *p*-NP palmitate (C16). For reactions using C4–C10 substrates (short- and medium-chain), the total reaction volume was 800 μL, consisting of 20 μL of 10 mM p-NP ester, 20 μL of ethanol, 740 μL of 50 mM Tris–HCl (pH 7.0), and 20 μL of recombinant esterase (0.1 mg/mL). For reactions using C12–C16 substrates (long-chain), the total reaction volume was 800 μL, consisting of 20 μL of 10 mM p-NP ester, 20 μL of ethanol, 740 μL of a mixture of 0.1% (*w*/*v*) gum arabic, 0.1% (*w*/*v*) sodium deoxycholate, and 50 mM Tris–HCl (pH 7.0), and 20 μL recombinant esterase (0.1 mg/mL). Reactions were incubated at the optimal temperature and pH for 15 min and immediately quenched on ice. Absorbance was measured at 405 nm (Molecular Devices, Sunnyvale, CA, USA). Activity corresponding to the highest absorbance was defined as 100%; all other values were expressed relative to this maximum. The unit (U) refers to the amount of esterase required to catalyze the formation of 1 μmol of pNP per minute under the same reaction conditions (temperature, pH, etc.).

### 2.8. Thermostability of the Esterase

The recombinant esterase was pre-incubated in a water bath set at 50–80 °C for 0–8 h. Immediately after heat treatment, residual activity was determined under the condition: the total reaction volume was 800 μL, containing 20 μL of 10 mM p-NP butyrate (C4), 20 μL of ethanol, 740 μL of 50 mM Tris–HCl (pH 7.0), and 20 μL of pre-heated enzyme (0.1 mg/mL). The reaction was initiated by transferring the mixture to 60 °C for exactly 15 min, then quenched on ice. Absorbance at 405 nm was recorded spectrophotometrically. The highest absorbance obtained at 0 h was defined as 100%, and all other activities were expressed relative to this maximum.

Fluorescence emission spectra of the enzyme were acquired under two thermal regimes. Temperature-gradient protocol: aliquots of recombinant esterase (0.1 mg/mL) were heated for 1 h at 50, 60, 70 or 80 °C, then immediately cooled on ice. Time-gradient protocol: aliquots were pre-incubated at 80 °C for 0, 2, 4, 6 or 8 h before detecting residual enzymatic activities. All samples were transferred to a black 96-well plate and scanned with a microplate reader (excitation: 280 nm, emission: 300–500 nm, slit widths: 4.66 nm for both excitation and emission) (Molecular Devices).

### 2.9. Effect of Various Reagents on the Esterase

The influence of various chemical factors on esterase activity was assessed spectrophotometrically containing 20 μL of 10 mM *p*-NP butyrate (C4), 20 μL of ethanol and 740 μL of 50 mM Tris–HCl buffer (pH 7.0), supplemented with 50 mM KCl, 50 mM CaCl_2_, 50 mM MgCl_2_, 50 mM NaCl, 0–10 mM EDTA, 0.1% (*w*/*v*) SDS or 0.1% (*v*/*v*) H_2_O_2_, respectively. After enzyme addition, the mixture was incubated at 60 °C for 15 min, immediately quenched on ice, and the absorbance at 405 nm was measured (Molecular Devices). The activity of the additive-free control was set to 100% and the relative activity of each treatment was calculated as the ratio of its absorbance to that of the control.

### 2.10. Data Statistical Analysis

All experimental data are presented as mean ± standard deviation. All of our experiments were performed in triplicate. Differences among group means were compared using Duncan’s multiple range test, with statistical significance set at a 95% confidence level (*p* < 0.05). This analysis was specifically conducted to assess the activity of Est1 with a variety of p-nitrophenyl esters as substrates. All statistical analyses were performed using IBM SPSS Statistics software, version 31 (SPSS Inc., Chicago, IL, USA).

### 2.11. Accession Numbers

The strain *L. sacchari* HS49-1 has been deposited at the Bioresource Collection and Research Center (BCRC) of the Food Industry Research and Development Institute in Taiwan, with the accession number BCRC AC10098. The whole-genome sequencing data of this strain have been deposited in GenBank under the accession number PRJNA1305130. Additionally, the Est1, Est2, and Est7 genes have been deposited in GenBank under the accession numbers PX121224, PX121225, and PX121226, respectively.

## 3. Results

### 3.1. Identification and Characterization of Strain HS49-1

**Figure 1:**
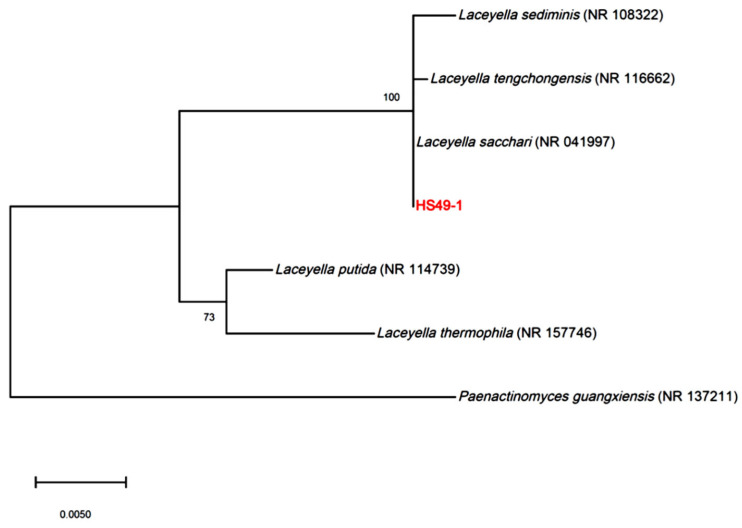
Phylogenetic tree based on 16S rDNA sequences, showing the evolutionary relationships of strain HS49-1 with species of the genus *Laceyella*, including *L. sediminis* (NR 108322), *L. tengchongensis* (NR 116662), *L. sacchari* (NR 041997), *L. putida* (NR 114739), and *L. thermophila* (NR 157746). *Paenactinomyces guangxiensis* (NR 137211) is included as an outgroup for comparative analysis to root the tree. Bootstrap values (based on 1000 replicates) are shown at the nodes, indicating the reliability of branching.

Strain HS49-1 was isolated from a hot spring source. Growth characterization revealed that the thermophilic strain exhibited an optimal growth temperature of 55 °C, with a viable growth range between 45 and 60 °C (Table S1). The strain demonstrated remarkable pH tolerance, growing across a broad pH range of 5 to 10, with optimal growth observed at pH 6–7 (Table S2). Enzyme activity profiling using the API ZYM system showed temperature-dependent enzymatic activities (Table S3). At 37 °C, only esterase (C4), esterase lipase (C8), and naphthol-AS-B1-phosphohydrolase displayed significant activity, while leucine arylamidase and α-chymotrypsin showed weak activity. When the reaction temperature was increased to 55 °C, multiple enzymes exhibited pronounced activity, including alkaline phosphatase, esterase (C4), esterase lipase (C8), leucine arylamidase, α-chymotrypsin, naphthol-AS-B1-phosphohydrolase, and α-glucosidase. Phylogenetic analysis based on 16S rRNA gene sequencing and NCBI database comparisons revealed that HS49-1 formed a distinct cluster with *L. sacchari*, *L. sediminis*, and *L. tengchongensis*, showing closest similarity to *L. sacchari* (Figure 1). This strain has been deposited in the Bioresource Collection and Research Center (Food Industry Research and Development Institute, Taiwan) under the designation *L. sacchari* BCRC AC10098.

### 3.2. Draft Genome Sequence of L. sacchari HS49-1

The genome of *L. sacchari* HS49-1 comprised 3,269,339 bp, which is relatively smaller among the compared *Laceyella* species (Table S4). Notably, *L. putida* possessed the largest genome at 4,075,529 bp. Strain HS49-1 contained 3350 predicted genes, a number that is moderate and commensurate with its genome size among the compared strains. The GC content of HS49-1 was determined to be 49%, which was comparable to other *Laceyella* species. Moreover, comparative analysis identified distinct differences in esterase and lipase gene content, with HS49-1 exhibiting a significantly higher number of annotated esterase genes. Functional annotation through GO analysis demonstrated enrichment in DNA-binding and oxidoreductase activities (molecular function), transmembrane transport and ribosome-related components (cellular component), and transmembrane transport processes (biological process) (Figure S1). COG classification revealed the most abundant functional categories to be general function prediction, followed by amino acid transport and metabolism, and inorganic ion transport and metabolism. KEGG pathway analysis indicated the strain’s involvement in multiple amino acid biosynthesis and degradation pathways (including alanine, phenylalanine, and tryptophan metabolism), carbohydrate biosynthesis and degradation, as well as key energy metabolic pathways.

### 3.3. Esterase Activity Screening Using Genomic Shotgun Library

**Figure 2:**
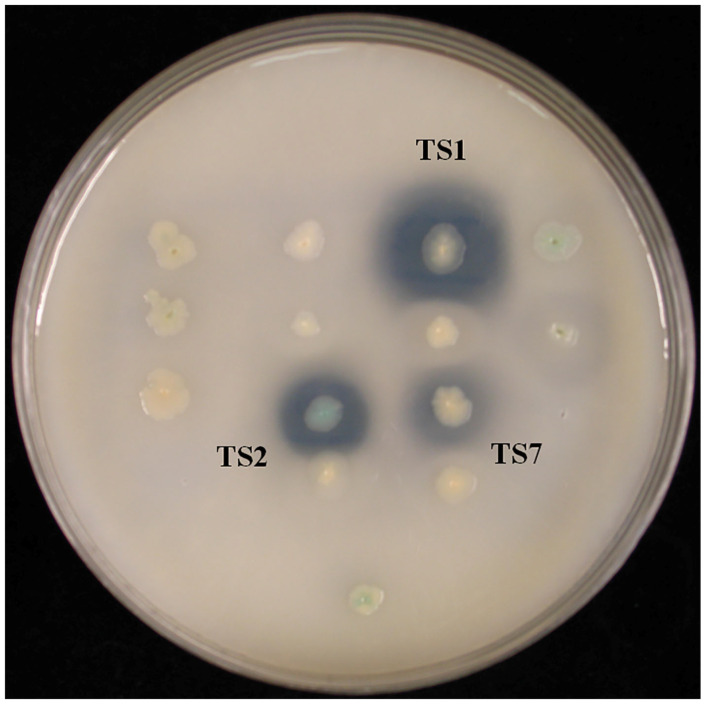
Transformants derived from the genomic shotgun library of *L. sacchari* HS49-1 were plated on LB agar supplemented with 1% triolein to assess esterase activity via clear-zone formation.

**Figure 3:**
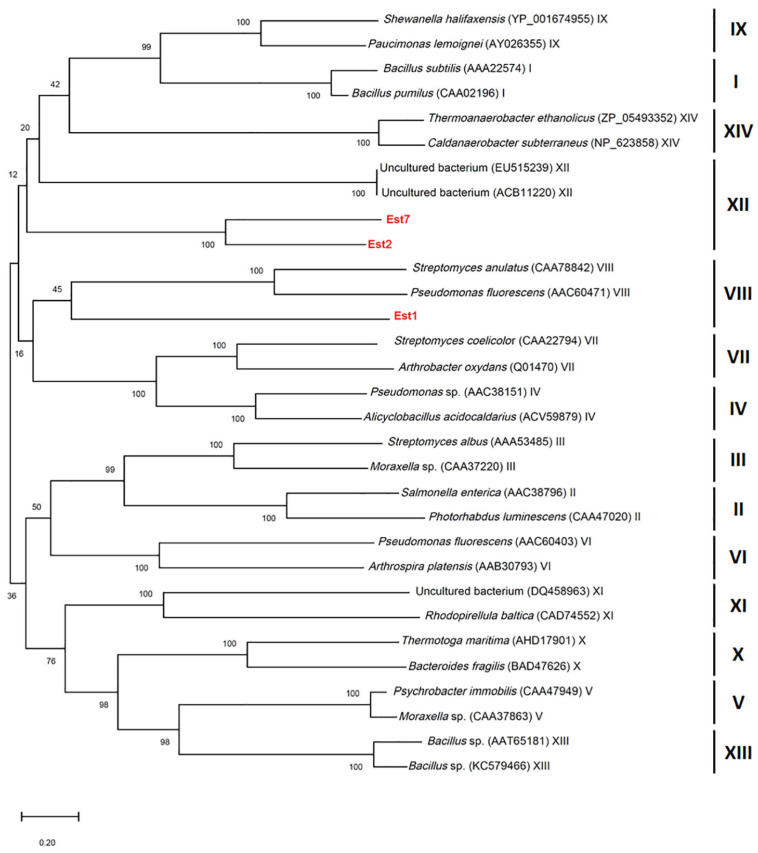
Phylogenetic tree constructed based on esterase sequences, showing the evolutionary relationships of Est1, Est2, and Est7 with esterases from various bacterial taxa and uncultured bacteria. Bootstrap values (based on 1000 replicates) are shown at the nodes, indicating the reliability of branching.

Chromosomal DNA from *L. sacchari* HS49-1 was sheared to generate 1–4 kb fragments and used to construct a genomic shotgun library. Transformants were plated on LB agar containing 1% triolein and screened for esterase activity based on clear-zone formation. Three clones—TS1, TS2 and TS7—displayed pronounced esterase activity (Figure 2). Notably, the TS1 clone produced the largest clear zone, indicating the strongest esterase activity among the three positive isolates. These three clones were selected for further DNA sequencing analysis. The results revealed open reading frames encoding proteins of 284, 523 and 537 amino acids, designated *Est1*, *Est2* and *Est7*, respectively. Phylogenetic comparison with characterized esterase families placed *Est1* in family VIII, while *Est2* and *Est7* clustered within family XII (Figure 3).

### 3.4. Construction and Overexpression of Recombinant Esterases in E. coli

**Figure 4:**
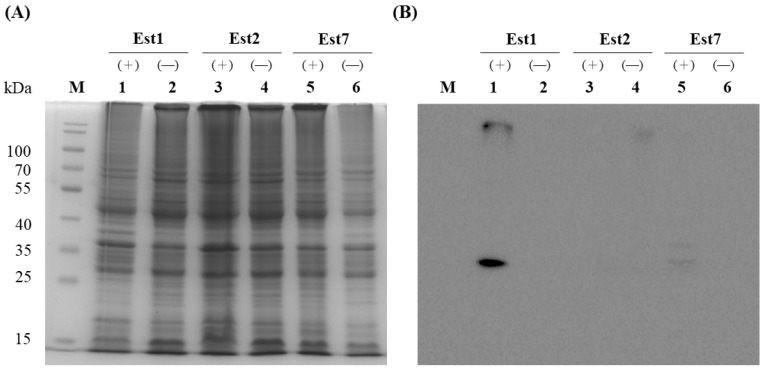
Overexpression of recombinant Est1, Est2, and Est7 in *E. coli* C43(DE3). (**A**) Coomassie blue-stained SDS-PAGE gel showing esterase proteins from soluble cell-free extracts, with lanes 1, 3, and 5 representing samples induced with 0.5 mM IPTG (+) and lanes 2, 4, and 6 representing uninduced controls (−). (**B**) Western blot analysis of esterase proteins from soluble cell-free extracts, with lanes 1, 3, and 5 corresponding to 0.5 mM IPTG-induced samples (+) and lanes 2, 4, and 6 to uninduced controls (−). M indicates the protein marker.

**Figure 5:**
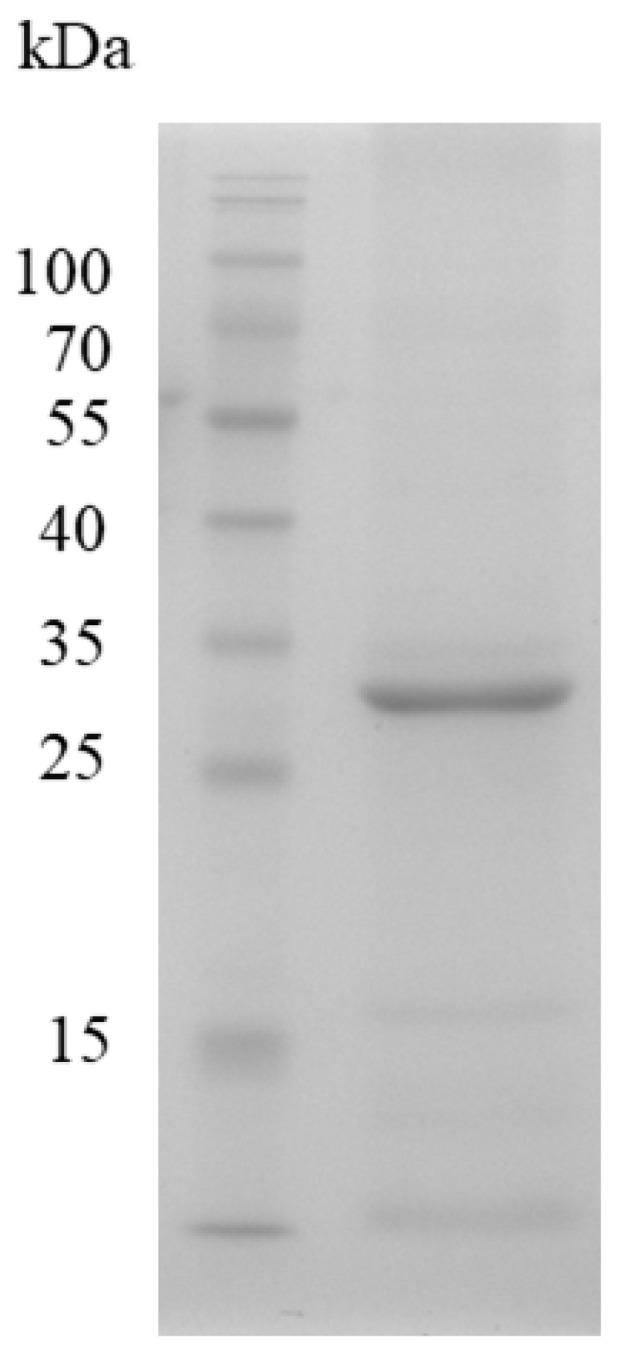
SDS-PAGE analysis of Est1 protein purification. A prominent band on the gel, observed after purification via a nickel column, indicates the Est1 protein. M indicates the protein marker.

To purify the three esterases, the corresponding genes were sub-cloned into the pET15b vector to yield N-terminal His-tagged constructs in *E. coli*. IPTG induction was used to drive expression, and the presence of each protein was verified by Western blotting with an anti-His antibody. SDS-PAGE analysis of whole-cell lysates revealed no discernible overexpression bands regardless of IPTG (Figure 4). However, Western blotting demonstrated a strong immunoreactive band exclusively for Est1 after IPTG induction; Est2 and Est7 remained undetectable. Consequently, Est1 was purified by Ni^2+^-NTA affinity chromatography, yielding a single dominant band on SDS-PAGE that matched the predicted molecular mass of 31.8 kDa (Figure 5). The purification process, utilizing a conical flask in conjunction with sonication and Ni^2+^-NTA affinity chromatography, achieved a yield of Est1 protein of 0.47 ± 0.09 mg/L.

### 3.5. Characterization of the Recombinant Esterase, Est1

**Figure 6:**
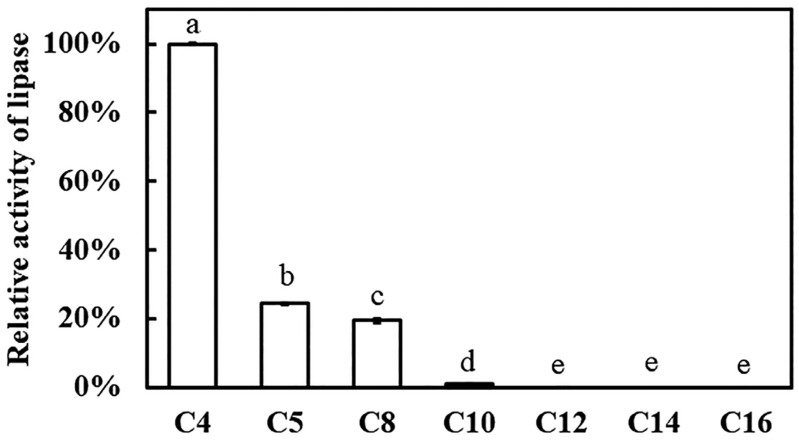
Evaluation of Est1 activity using various *p*-nitrophenyl esters as substrates. Relative activity was calculated with the highest activity set as 100%. Reactions were performed at pH 7.0 and 60 °C for 15 min, followed by analysis of absorbance at 405 nm. Different letters indicate significant differences among groups.

Using *p*-nitrophenyl (*p*-NP) esters as substrates, esterase activity was determined spectrophotometrically. The catalytic profile of recombinant Est1 was then systematically investigated with a homologous series of *p*-NP esters: *p*-NP butyrate (C4), *p*-NP valerate (C5), *p*-NP caprylate (C8), *p*-NP caprate (C10), *p*-NP laurate (C12), *p*-NP myristate (C14), and *p*-NP palmitate (C16). Recombinant Est1 displayed robust hydrolytic activity toward short-chain substrates—namely p-NP butyrate (C4), p-NP valerate (C5), and p-NP caprylate (C8)—with a marked preference for p-NP butyrate (C4) (Figure 6). Based on the analysis of enzyme activity, the results indicate that Est1 exhibited activities of 57.43 ± 0.04, 13.00 ± 0.15, and 10.08 ± 0.26 U/mg towards p-NP butyrate (C4), p-NP valerate (C5), and p-NP octanoate (C8), respectively.

**Figure 7:**
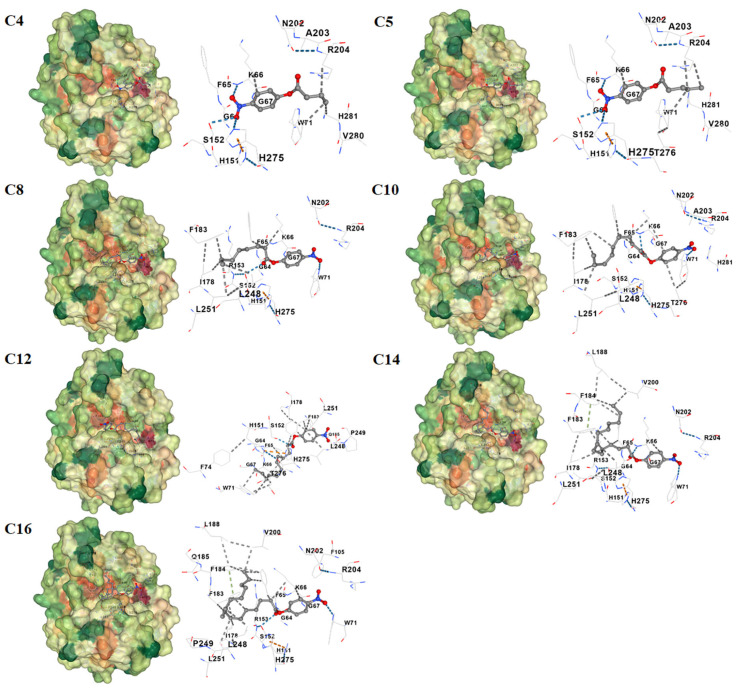
Molecular docking analysis of Est1 with various p-nitrophenyl esters. The three-dimensional structure of Est1 was predicted via SWISS-MODEL homology modeling, and molecular docking was performed using CD-Dock2 (https://cadd.labshare.cn/cb-dock2/index.php) (accessed on 25 June 2025). The dashed lines in blue, grey, orange, and green correspond to hydrogen bonds, hydrophobic interactions, π–cation interactions, and π–π stacking, respectively.

To rationalize these findings, molecular-docking analyses were performed between recombinant Est1 and each p-NP ester (Figure 7). The three-dimensional structure of Est1 was predicted using SWISS-MODEL homology modeling [27]. The conserved GXSXG catalytic motif has also been identified in the Est1 [28], and all these various substrates were indeed found to interact with Est1’s catalytic motif (specifically at S152) [29]. As the acyl chain lengthened, the calculated binding energy progressively decreased, accompanied by an increased number of hydrophobic contacts (Table 1). Notably, the longest substrates, p-NP myristate (C14) and p-NP palmitate (C16), established additional π–π stacking interactions. In contrast, the shortest substrate, p-NP butyrate (C4), formed only one π-cation interaction, five hydrogen bonds, and four hydrophobic interactions with the enzyme, underscoring the distinct binding mode.

**Figure 8:**
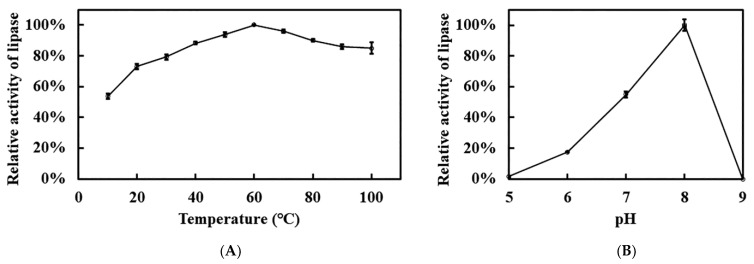
Optimal temperature and pH analyses of Est1. (**A**) Temperature profile of Est1, determined at 10–100 °C under pH 7 for 15 min. (**B**) pH profile of Est1, assessed at 60 °C using 50 mM sodium acetate/acetic acid buffer (pH 5), 100 mM sodium phosphate buffer (pH 6–8), and glycine/NaOH buffer (pH 9) for 15 min.

Due to the thermophile *L. sacchari* HS49-1, recombinant Est1 was profiled across a broad temperature and pH spectrum to define its optimal catalytic activity (Figure 8). The enzyme peaked at 60 °C yet exhibited extraordinary thermostability. The decline in activity beyond the optimum was gradual, retaining 85% of its maximal rate at 100 °C. Remarkably, even at 10 °C recombinant Est1 preserved 53.5% relative activity, underscoring its broad thermal adaptability. In contrast to this pronounced temperature tolerance, recombinant Est1 displayed a markedly narrower pH range. Activity was maximal at pH 8.0, whereas values below 6.0 or above 8.0 led to almost complete loss of function, revealing stringent requirements for mildly alkaline conditions.

### 3.6. Thermostable Assay of the Recombinant Esterase, Est1

**Figure 9:**
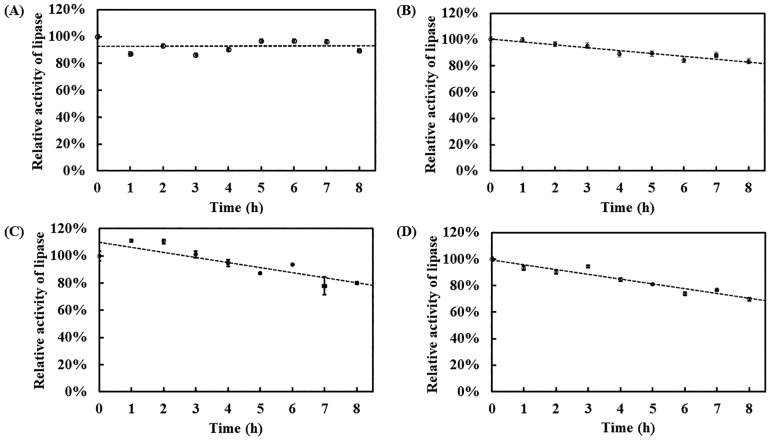
Thermostability analysis of Est1 at various temperatures. Est1 was incubated at (**A**) 50 °C, (**B**) 60 °C, (**C**) 70 °C, and (**D**) 80 °C for 8 h. Residual enzyme activity was measured at 60 °C for 15 min, followed by absorbance analysis at 405 nm. The circles represent the residual activity of Est1 after being subjected to various temperatures for different durations. The line illustrates the trend over time following these treatments.

To assess the long-term thermostability of recombinant Est1, aliquots were pre-incubated at 50–80 °C for up to 8 h and residual activity was subsequently determined. After 8 h of pre-incubation at 50 °C, the enzyme retained 90% of its initial activity (Figure 9). As the pre-incubation temperature rose, a gradual decline in stability was observed. Relative activities of 83.6%, 80.2%, and 69.8% remained after 8 h at 60 °C, 70 °C, and 80 °C, respectively. Notably, within the 50–80 °C, recombinant Est1 maintained over 90% relative activity for at least the first 2 h, underscoring its robust thermal stability and suitability for high-temperature applications.

**Figure 10:**
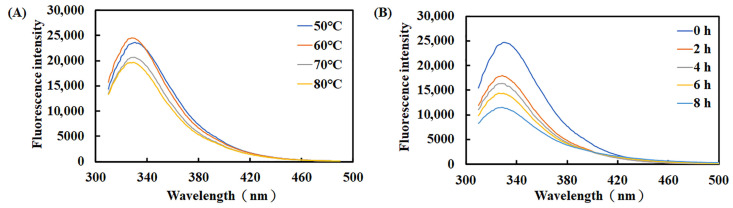
Fluorescence spectra of Est1 following treatment at different temperatures and durations. (**A**) Est1 was incubated at 50–80 °C for 1 h. (**B**) Est1 was incubated at 80 °C for 8 h. Fluorescence spectra were recorded using a microplate reader.

According to the intrinsic fluorescence of tryptophan, tyrosine, and phenylalanine, we examined how temperature and incubation time affected the tertiary structure of recombinant Est1. The native enzyme exhibited an average maximum emission wavelength of 328–331 nm (Figure 10). Pre-incubation at 50 °C or 60 °C for 1 h left the fluorescence spectrum unchanged, indicating preserved conformation. Raising the temperature to 70 °C or 80 °C reduced the fluorescence intensity by only 12.1% and 16.6%, respectively, revealing modest short-term perturbations. To assess long-term thermal stress, recombinant Est1 was pre-treated at 80 °C for 0–8 h. Fluorescence measurements revealed a gradual decline in intensity, ultimately reaching a 53% reduction after 8 h of observation. This pronounced quenching demonstrates that prolonged high-temperature exposure substantially disrupts the protein structure.

### 3.7. Impact of Chemical Reagents on the Recombinant Esterase, Est1, Activity

**Figure 11:**
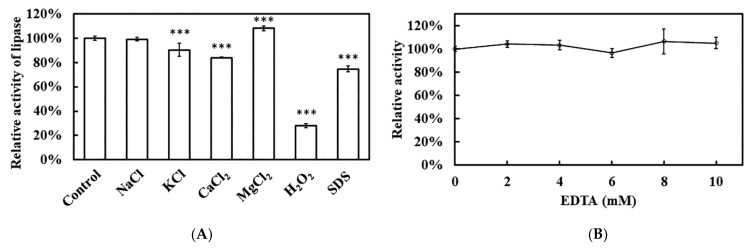
Effect of various reagents on Est1 activity. (**A**) Different reagents (50 mM NaCl, KCl, CaCl_2_, MgCl_2_; 0.1% H_2_O_2_, and SDS) and (**B**) EDTA (0–10 mM) were added to assess Est1 activity at 60 °C for 15 min. Residual enzyme activity was determined via absorbance analysis at 405 nm. *** indicates a significant difference compared with the control group.

The influence of various chemical reagents and metal ions on recombinant Est1 activity was showed in Figure 11. Among the additives tested, H_2_O_2_ exerted the strongest inhibitory effect, followed by the anionic surfactant SDS. Among the metal ions, Ca^2+^ produced a notable change in activity. To clarify whether Est1 requires a metal cofactor, the enzymatic activity with increasing concentrations of EDTA was performed. Even at 10 mM EDTA, activity remained essentially unchanged, indicating that Est1 catalysis proceeds without the assistance of bound metal ions.

## 4. Discussion

The isolated strain, based on 16S rDNA sequencing and genomic data, revealed high similarity to *L. sacchari*. Comparative studies showed *L. sacchari* HS49-1 had a relatively narrow growth temperature range (45–60 °C) compared to other *Laceyella* type strains: *L. sacchari* KCTC 9790^T^ (35–65 °C), *L. putida* KCTC 3666^T^ (30–65 °C), *L. sediminis* RHA1^T^ (28–65 °C), *L. tengchongensis* YIM 10002^T^ (28–70 °C), and *L. thermophila* YIM 79486^T^ (37–65 °C) [10,11,12,13]. Like other members of this genus, it tolerated a broad pH range while preferring neutral conditions for optimal growth [10,11,12]. API ZYM assays detected significant esterase activity in *L. sacchari* HS49-1 at 37 °C, with additional enzymatic activities such as alkaline phosphatase and α-glucosidase emerging at 55 °C, indicating the presence of thermostable enzymes. As no systematic studies of *Laceyella* esterases have been reported previously, this study employed genome mining to identify and characterize novel esterases in this thermophilic genus.

In this study, we first constructed a genomic shotgun library of *L. sacchari* HS49-1 and conducted plate-based screening for esterase activity. Three distinct clones were identified, each forming clear zones on triolein plates—an indication of lipolytic activity. The three esterase gene clones (*Est1*, *Est2*, and *Est7*) from *L. sacchari* HS49-1 demonstrated high sequence conservation within the genus, with α/β hydrolase gene sequences showing 71.4–100% similarity among different *Laceyella* species (*L. tengchongensis*, *L. sacchari*, and *L. putida*) according to the NCBI BLAST (https://blast.ncbi.nlm.nih.gov/Blast.cgi, accessed on 8 July 2025). In contrast, when compared to esterases from other genera within the same family Thermoactinomycetaceae, including *Thermoactinomyces* (WP_170151265.1), *Salinithrix* (WP_380701314.1), and *Paenactinomyces* (WP_181752047.1), the sequence similarity dropped significantly to 51.32–64.52%. This substantial reduction in similarity highlighted the unique characteristics of *Laceyella*’s esterase genes, suggesting they have evolved distinct structural or functional features that differentiate them from related genera in the Thermoactinomycetaceae family.

Nevertheless, when these genes were subcloned into pET15b expression vectors driven by the T7 promoter, only the family VIII esterase, Est1, was successfully expressed. While factors such as codon optimization, fusion tags, vector selection, and promoter strength are known to influence protein expression [30], our analysis revealed no obvious inclusion body formation, suggesting the problem likely occurs during the translation process. These results implies that although the T7 promoter system is highly effective for recombinant protein production, it may be unsuitable for certain genes—particularly family XII esterase genes, which appear sensitive to the strong transcriptional activity of T7 promoters. This sensitivity could create bottlenecks during transcription or translation [31]. Additionally, the strong transcriptional activity driven by T7 promoters may lead to more complex mRNA secondary structures, particularly in the translation initiation region. These intricate structural conformations could potentially impede ribosomal binding and subsequent assembly, thereby significantly compromising translation initiation efficiency [32].

Analysis of Est1 activity toward p-nitrophenyl substrates with varying chain lengths demonstrated that Est1 exhibited optimal catalytic efficiency for the short-chain *p*-NP butyrate (C4), with activity progressively declining as the carbon chain length increases. Molecular docking studies revealed that while all substrates form essential hydrogen bonds with the enzyme, longer-chain substrates develop increasingly extensive hydrophobic interactions with Est1’s binding pockets. These findings indicate that substrate binding was stabilized through a combination of hydrogen bonding and hydrophobic interactions, both crucial for forming the enzyme-substrate complex and facilitating catalysis [33]. However, the enhanced hydrophobic interactions observed with longer-chain substrates, although strengthening binding affinity, appear to hinder product release, thereby reducing overall catalytic efficiency [34]. This trade-off between binding stability and catalytic turnover explained the inverse correlation observed between substrate chain length and enzymatic activity, highlighting the delicate balance required between substrate binding and product release in enzymatic catalysis.

Thermostable esterases often exhibit increased hydrophobicity in their protein sequences [35]. This elevated proportion of hydrophobic amino acids reinforces the protein’s hydrophobic core, thereby improving structural stability at high temperatures. Additional stabilizing factors include the formation of salt bridges (e.g., between Asp-Lys or Glu-Arg pairs) [36], an increased presence of secondary structural elements (α-helices and β-sheets) [37,38], and the formation of disulfide bonds, which collectively mitigate thermal denaturation [39]. Furthermore, specific residues such as proline, contribute to stability due to the rigidity of their side chains [40]. To investigate these mechanisms, we compared several Family VIII esterases, including PBS-2 (*Paenibacillus* sp.) [41], Est13L, DLFae4, and EstCS3 (metagenome-derived) [42,43,44], with optimal activity temperatures of 30 °C, 40 °C, 50 °C, and 55 °C, respectively. Notably, the thermostability of Est1 (60 °C) surpassed all these homologs (Table S5). Secondary structure prediction via YASPIN revealed that Est1 did not exhibit the highest number of α-helices or β-strands [45], suggesting that its thermostability is not primarily driven by these features. Instead, Est1 displayed a significantly higher proline content. Comparative analysis of hydrophobic (AILFWV) and charged (RKHYCDE) amino acids highlighted that Est1 and DLFae4 possessed the highest hydrophobic content, with phenylalanine, and valine being major contributors. These findings suggest that thermostability in Family VIII esterases is predominantly achieved through enrichment of hydrophobic residues, with Est1 serving as a striking example of this trend.

The relative activity of the esterase under various treatments revealed distinct responses to ions, oxidizing agents, and detergents. Notably, NaCl, KCl, CaCl_2_, and MgCl_2_ had minimal impact on enzyme activity, with values remaining close to the control. This suggests that the esterase was highly tolerant to monovalent (Na^+^, K^+^) and divalent (Ca^2+^, Mg^2+^) cations, which may stabilize its structure or had negligible interference with the active site. In contrast, H_2_O_2_ and SDS significantly reduced activity, with SDS showing a more pronounced inhibitory effect. The sensitivity to H_2_O_2_ implies that oxidative modification (e.g., of catalytic residues) may disrupt function [46], while the strong inhibition by SDS likely reflects protein denaturation due to disruption of hydrophobic interactions critical for stability [47]. Critically, EDTA had no inhibitory effect across the tested range (0–10 mM), as activity remained unchanged compared to the control. This confirms that the esterase does not require metal cofactors for catalysis or structural stability, distinguishing it from metalloenzymes [48]. The absence of EDTA sensitivity aligns with its tolerance to ionic treatments, reinforcing its metal-independent mechanism.

## 5. Conclusions

We have identified and characterized a novel esterase, named Est1 and classified under family VIII, originating from the thermophilic bacterium *L. sacchari* HS49-1—a relatively understudied strain. Genomic analysis revealed significant divergence between the genes of this bacterium and those of its congeners, underscoring the distinctiveness of this genus. Our successful heterologous expression of Est1 in *E. coli* yielded an enzyme with outstanding thermostability, maintaining 69.8% of its activity after an 8-h incubation at 80 °C. Furthermore, Est1 functioned independently of metal ion cofactors, making its activity less susceptible to interference from metal ions in industrial applications. The remarkable thermal resilience of Est1 highlighted the unique adaptive features of *Laceyella*-derived enzymes and positioned it as a promising candidate for industrial processes requiring robust biocatalysts under extreme temperatures.

## Figures and Tables

**Table 1:** Molecular docking simulations were conducted using CB-Dock2 to systematically assess the binding interactions between Est1 and a series of substrates with varying acyl-chain lengths (C4-C16).

Substrates	Binding Energy (kcal/mol)	π–π	π–Cation	H-Bond	Hydrophobic Interaction
C4	−6.2	0	1	5	4
C5	−6.0	0	1	5	5
C8	−6.3	0	1	5	8
C10	−6.2	0	1	4	12
C12	−6.6	0	1	2	15
C14	−6.5	1	1	5	14
C16	−6.6	1	1	5	17

## Data Availability

The raw data supporting the conclusions of this article will be made available by the authors on request.

## Supplementary Materials

The following supporting information can be downloaded at: https://www.mdpi.com/article/10.3390/genes16111330/s1, Figure S1: Genomic analysis of *L. sacchari* H49-1; Table S1: The growth characteristics of strain HS49-1 were observed by culturing it in YM medium at various temperatures; Table S2: The growth characteristics of strain HS49-1 were observed by culturing it in YM medium at various pH levels; Table S3: The enzymatic activities of strain HS49-1 were analyzed using the API ZYM system at both 37 °C and 55 °C; Table S4: Genome sequence summary of *L. sacchari* H49-1 and published *Laceyella* species; Table S5: A comparison of optimal temperature, pH, protein secondary structure, and amino acid sequence composition between Est1 and other proteins belonging to the same family VIII esterases.
