# Supplementary material for: Thermostable Esterase from Thermophilic Laceyella sacchari: Gene Identification, Heterologous Expression, and Biocatalytic Characterization
Source: Genes (Basel). 2025 Nov 3;16(11):1330. doi: 10.3390/genes16111330 (PMC12652962; doi:10.3390/genes16111330)
Supplement: Supplementary file 1 [file genes-16-01330-s001.zip › genes-3943884-supplementary.pdf]

Table S1. The growth characteristics of strain HS49-1 were observed by culturing it in YM medium at various temperatures.

Temp. (℃)	20	25	30	37	45	50	55	60	65
Growth	—	—	—	—	+	++	+++	+	—

Growth intensity was classified as: '+++' (vigorous), '++' (moderate), '+' (mild), or '-' (absent).

Table S2. The growth characteristics of strain HS49-1 were observed by culturing it in YM medium at various pH levels.

pH	4	5	6	7	8	9	10
Growth	—	++	+++	+++	+	+	W

Growth intensity was classified as: '+++' (vigorous), '++' (moderate), '+' (mild), 'W' (weak but present), or '-' (absent).

Table S3. The enzymatic activities of strain HS49-1 were analyzed using the API ZYM system at both 37°C and 55°C.

API ZYM	37℃	55℃
Alkaline phosphatase	—	+
Esterase(C4)	+	+
Esterase lipase (C8)	+	+
Lipase (C14)	—	—
Leucine arylamidase	W	+
Valine arylamidase	—	—
Cystine arylamidase	—	—
Trypsin	—	—
α-Chymotrypsin	W	+
Acid phosphatase	—	—
Naphthol-AS-B1-phosphohydrolase	+	+
α-Galactosidase	—	—
β-Galactosidase	—	—
β-Glucuronidase	—	—
α-Glucosidase	—	+
β-Glucosidase	—	—
N-Acetyl-β-glucosaminidase	—	—
α-Mannosidasea	—	—
α-Fucosidase	—	—

Enzymatic activity was classified as: '+' (clearly positive), 'W' (faintly positive), or '-' (negative), based on visual intensity of color development.

Table S4. Genome sequence summary of *Laceyella sacchari* H49-1 and published *Laceyella* species.

Category	H49-1	*L. sacchari*	*L. sediminis*	*L. tengchongensis*	*Laceyella putida*
Total Number of Contigs	93	2	44	31	92
Genome size (bp)	3,269,339	3,383,707	3,392,166	3,352,889	4,075,529
Gene annotation	3,350	3,331	3,460	3,443	4,187
GC%	49	48.5	49	49	48.5
Esterase	35	23	24	25	26
Lipase	6	8	6	7	8
BioSample ID	This study	SAMN30556255	SAMN07621173	SAMN06265361	SAMN43283147

Table S5. A comparison of optimal temperature, pH, protein secondary structure, and amino acid sequence composition between Est1 and other proteins belonging to the same family VIII esterases.

Terms	Est1	EstCS3	DLFae4	Est13L	PBS-2
Opt. Temp	60℃	55℃	50℃	40℃	30℃
Opt. pH	8	8	8.6	10	9
α helix	8	11	10	13	11
β strand	8	9	7	9	10
Proline (%)	4.93	3.91	5.03	4.87	4.51
Hydrophobic A.A. (%)	37.68	34.23	37.28	35.04	32.89
Charged A.A. (%)	29.23	28.85	23.96	28.22	29.71
Alanine (%)	8.45	10.51	10.36	9.00	7.16
Phenylalanine (%)	5.99	4.16	5.33	5.35	3.71
Valine (%)	5.99	6.85	7.69	5.11	5.84

(A)

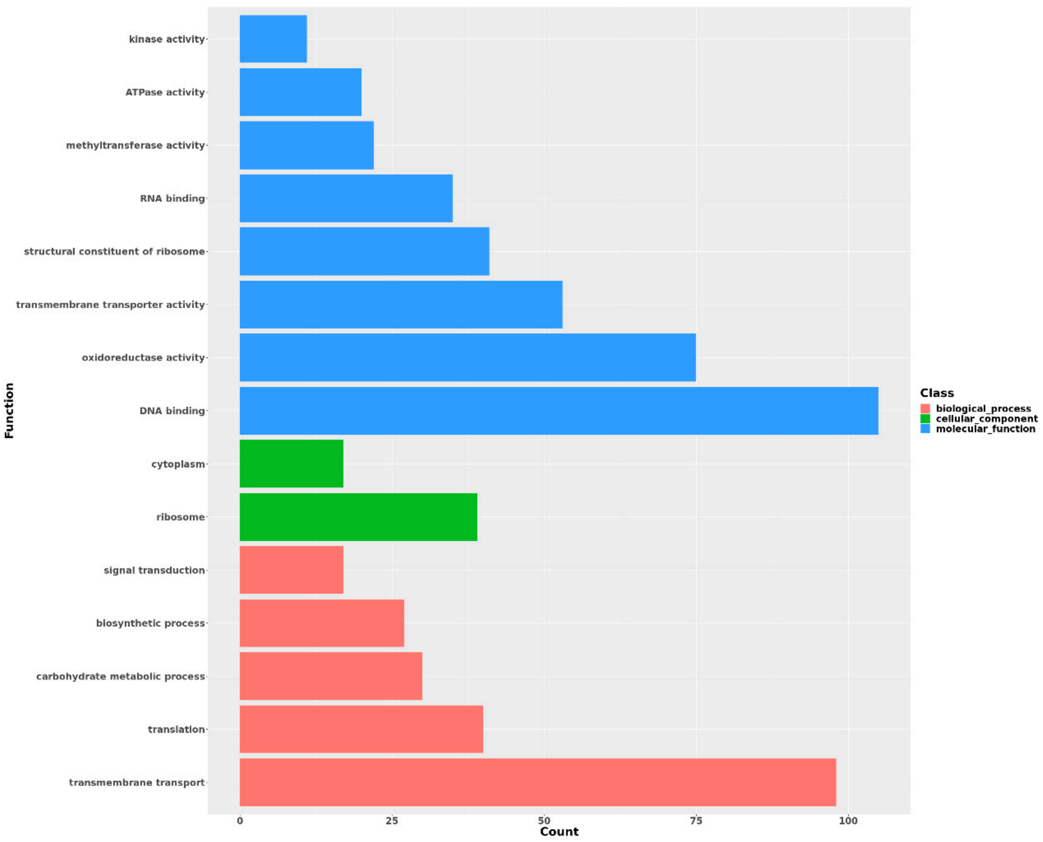
kinase activity
ATPase activity
methyltransferase activity
RNA binding
structural constituent of ribosome
transmembrane transporter activity
oxidoreductase activity
DNA binding
cytoplasm
ribosome
signal transduction
biosynthetic process
carbohydrate metabolic process
translation
transmembrane transport
Function
0
25
50
75
100
Count
Class
biological_process
cellular_component
molecular_function

(B)

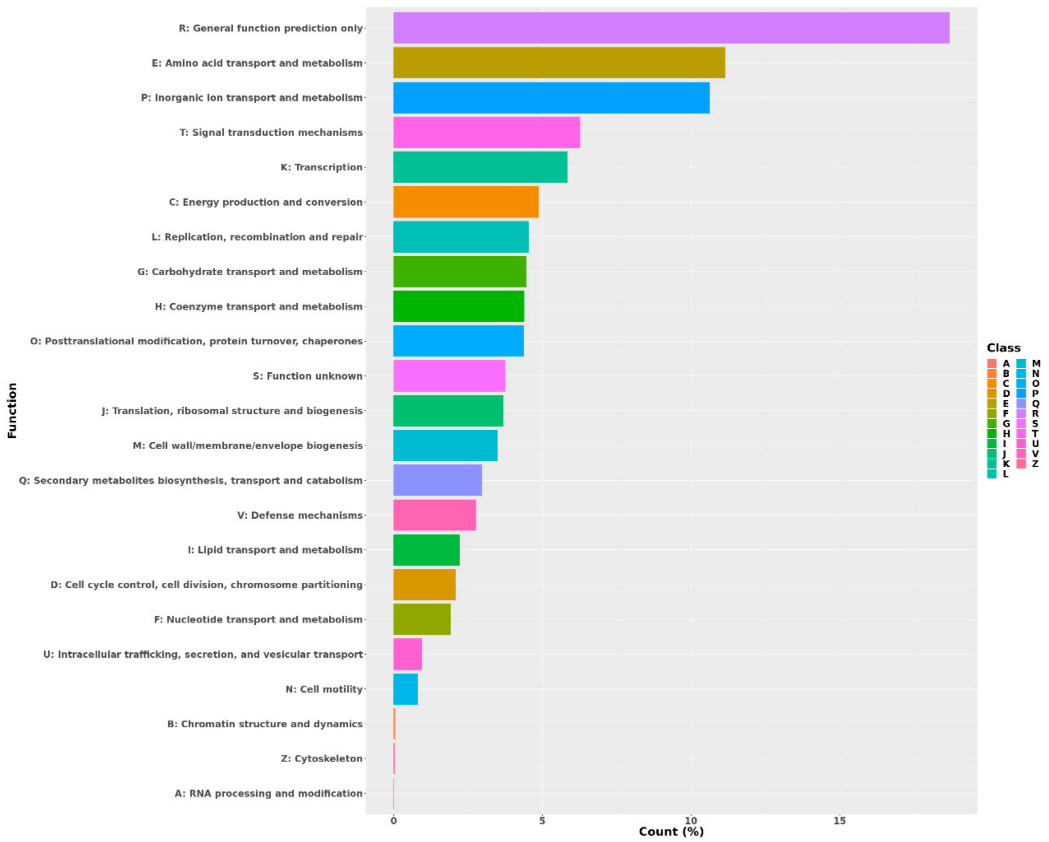

R: General function prediction only
E: Amino acid transport and metabolism
P: Inorganic ion transport and metabolism
T: Signal transduction mechanisms
K: Transcription
C: Energy production and conversion
L: Replication, recombination and repair
G: Carbohydrate transport and metabolism
H: Coenzyme transport and metabolism
O: Posttranslational modification, protein turnover, chaperones
S: Function unknown
J: Translation, ribosomal structure and biogenesis
M: Cell wall/membrane/envelope biogenesis
Q: Secondary metabolites biosynthesis, transport and catabolism
V: Defense mechanisms
I: Lipid transport and metabolism
D: Cell cycle control, cell division, chromosome partitioning
F: Nucleotide transport and metabolism
U: Intracellular trafficking, secretion, and vesicular transport
N: Cell motility
B: Chromatin structure and dynamics
Z: Cytoskeleton
A: RNA processing and modification
Function
0
5
10
15
Count (%)
Class
A
B
C
D
E
F
G
H
I
J
K
L
M
N
O
P
Q
R
S
T
U
V
Z

(C)

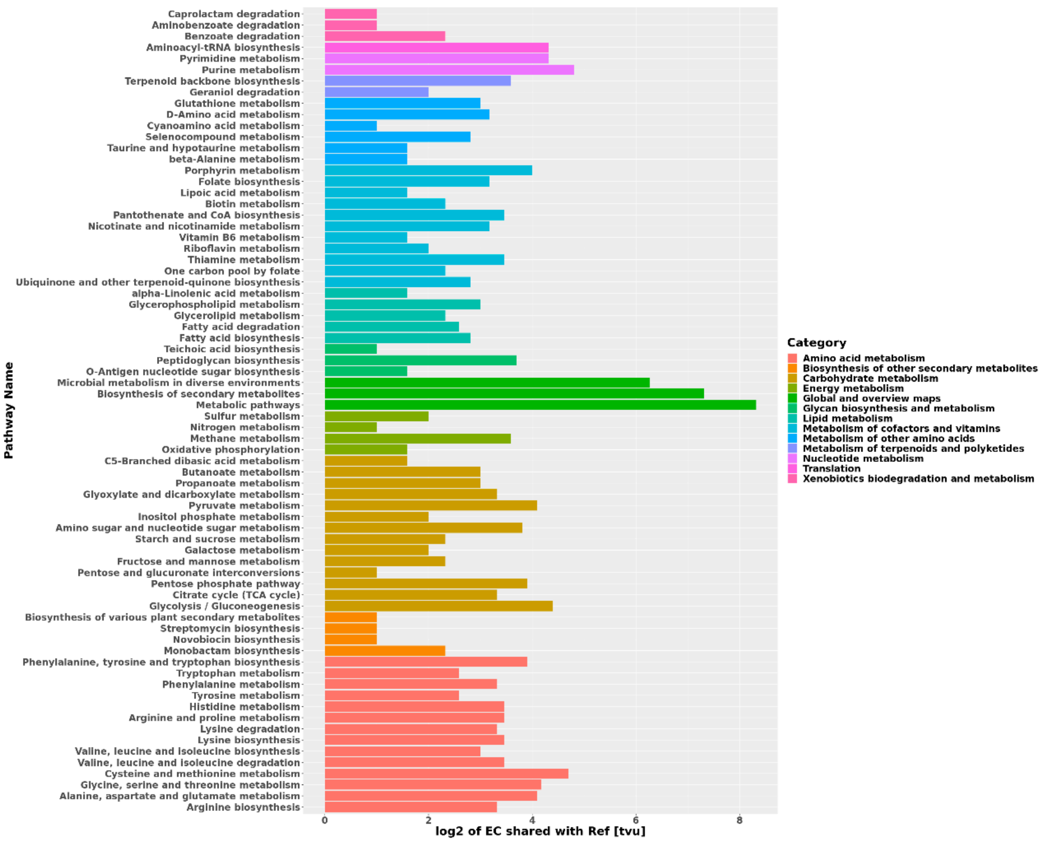

Figure S1. Genomic analysis of *L. sacchari* H49-1. Gene annotation results based on (A) Gene Ontology (GO), (B) Clusters of Orthologous Groups (COG), and (C) Kyoto Encyclopedia of Genes and Genomes (KEGG) databases.
